# Weight estimation in Paediatrics: how accurate is the Broselow-tape weight estimation in the Nigerian child

**DOI:** 10.1186/s13052-019-0744-5

**Published:** 2019-11-19

**Authors:** Ogochukwu N. Iloh, Benedict Edelu, Kenechukwu K. Iloh, Obianuju O. Igbokwe, Ikenna K. Ndu, Obinna C. Nduagubam, Uzoamaka C. Akubuilo, Ijeoma N. Obumneme-Anyim, Joy N. Eze, Chidiebere D. I. Osuorah

**Affiliations:** 1 University of Nigeria Teaching Hospital, Nsukka, Nigeria; 2 Enugu State University of Science and Technology, Enugu, Enugu State Nigeria; 3 Child Survival Unit, Medical Research Council UK, The Gambia Unit, Fajara, The Gambia

**Keywords:** Weight, Broselow tape, Children, Accuracy

## Abstract

**Background:**

Determination of weight in children is an important aspect of their assessment. It has a wide range of usefulness including assessing their nutritional status and drug dose calculation. Despite its usefulness, weight estimation in children in certain conditions can be challenging particularly in emergency situations or in children who are severely ill or cannot stand on standard scales. The Broselow Tape which is a validated tape that is used to estimate weight based on length was developed using height/weight correlations from Western data. However, considering the variations in anthropometric measurements of children from different geographic locations, there is need to ascertain how accurate it is to estimate weight using the Broselow tape among children in Nigeria.

**Aim:**

The study was carried out to determine the accuracy in the use of the Broselow Tape in weight estimation among Nigerian children.

**Method:**

A total 1456 children aged 1–12 years who satisfied the inclusion criteria were enrolled over a 2½ year period from two tertiary health facilities in Enugu state Nigeria. Weight was taken using standard weighing scale and Broselow tape. Data collected was analysed using SPSS.

**Result:**

Of the 1456 children studied, majority (84.2%) had normal Body-Mass-Index (BMI) while about 4.6% had a low BMI percentile for age. The mean weight difference between the two methods was not significantly different between the 1 to 6 years old category. Significant differences were observed from 7 up to 12 years. The Broselow Tape overestimated weights in 1 year old by 3.88%, 2 years 1.58%, 3 years by 2.13%, 4 years (1.94%) and 5 year (0.07%). After 5 years, the degree of overestimation rises sharply to 4.25% in 6, 9.25% in 7, 7.29% in 8 and 9.29%. 9.18, 11.61% & 6.75% in 9, 10, 11 and 12 years old respectively. The proportion of estimated weights that was within 10- 20% of the actual weight was higher in the 1-6 years age categories compared to weight estimates in older age categories.

**Conclusion:**

Weight estimates obtained using the Broselow tape correlated better in children that are 6 years or younger compared to those in the older age categories. There is need for re-validation and/or adjustments of the Broselow tape especially in children over 6 years old.

## Introduction

The determination of the weight of a child is an essential part of paediatric practice whether in the emergency unit, ward or clinic setting. The weight is an important element in making a number of diagnostic and treatment decisions including nutritional status assessment, calculating drug doses, sizes of equipment, use of treatment normograms, fluid therapy and energy levels for defibrillation [1, 2]. Weight determination is a major component of growth monitoring and it is critical to the institution of most preventive child health interventions included in the child survival strategies [3]. Also, in paediatric emergencies, drug doses and intervention decisions are often based on estimated body weights [4].

Calculating, prescribing, preparing, and delivering accurate drug doses to children in the emergency department can be challenging at the best of times and consequently medication errors in children have been shown to be very common [5]. The additional emotional stress and cognitive load of a paediatric resuscitation make the risk of incurring errors even higher [6, 7] and these errors are more likely to result in adverse events (patient harm) in the very young patient and the critically ill or injured child or infant with significant physiological insult [8]. Inaccurate weight estimations may cause non-responsiveness or increased adverse events and toxicity to interventions [5].

The most accurate method of determining a child’s weight is to weigh the child on a standard machine with calibrated scales, ‘the gold standard’. If possible, all children should be weighed on a scale to obtain their actual weight. However, this is often impossible or impractical in an emergency setting, and an accurate, rapid weight-estimation method is essential in these situations [9]. Over the past three decades, many different weight-estimation methods have been used, each with its own advantages and limitations. The most commonly used methods include guesses or estimations by parents or healthcare workers, age-based formulae and length-based methods.

The Broselow Tape which is commonly used in our setting was developed using height/weight correlations from Western data. This validated tape estimates weight (hence intervention doses/sizes) of the supine child based on length [10, 11]. The tape has a series of color-coded zones (boxes) with details of all necessary doses and sizes for emergency interventions. One end of the tape has a red arrow and this end is placed at the head end of the supine child with the tape lying alongside the child. The foot end of the child indicates the colored box with printed out estimated doses/sizes of fluids, drugs and equipment. This eliminates memorization and mathematical errors and one can concentrate more on administering emergency care. The Broselow tape is designed to estimate the body weight, medication doses and endotracheal tube sizes based on body length among children aged 1 to 12 years old. The aim of this study was to compare the actual recorded weights of Nigerian children to the predicted weights using Broselow tape.

## Methodology

### Study area

This cross-sectional multi-center study was conducted concurrently over a 2½ years period between January 2017 and May 2019 in two tertiary health facilities namely the University of Nigeria Teaching Hospital (UNTH) and the Enugu State University Teaching Hospital (ESUTH), both in Enugu state. Each of these hospitals offer specialized medical services and serves as a referral center to primary, secondary and private health facilities from within and outside Enugu state.

### Study participants

Children aged 1  to 12 years that presented to the emergency room and out-patient clinic were consecutively enrolled into the study. Excluded were children whose parents/caregiver refused to give consent and/or whom informed consent could not be reasonably given because of their child’s medical condition. Also excluded from this study were children too short (< 46 cm) or too tall (> 142 cm) to fit within the limits of the Broselow tape; children with serious medical conditions such that an actual weight cannot be measured and children with any medical condition (such as amputation, dehydration, edema, growth hormone deficiency, severe joint contractures, or neurologic defects) which could substantially affect their weight and/or height. Participation was entirely voluntary without any form of inducement. Parents/caregivers were informed that they were free to withdraw their child from the study at any time and with no adverse effect on their treatment or hospital visit. All subjects who met the inclusion criteria were consecutively enrolled.

### Measures

The children’s actual weight was measured using the Omron digital scale (HN-289-EB) for children ≥2 years old. For those < 2 years old, an infant weighing scale was used. The study participants were weighed wearing nothing but light clothing and no footwear. Weights were rounded off to nearest 0.1 kg. Length was measured using the latest version of the Broselow® Tape (Broselow® Paediatric Emergency Tape, 2017 Edition A, Drs. James Broselow and Robert Luten, Armstrong Medical Industries, 575 Knightsbridge Parkway, Lincolnshire, IL 60069, USA) which incorporated adjusted length-weight classes based on National Health and Nutrition Examination Survey (NHANES) data from the United States. The study participant were placed in supine position with the head kept in neutral position with no pillow in place. The end of the tape marked, “*measure from this end*” was positioned at the vertex and the tape stretched to the child’s heel with the hip and knee straight and the ankle flexed at 90°. The child’s weight was read off the tape at the point where it crossed the child’s heel.

Information on children’s basic socio-demographic characteristics were also obtained. These included i*)* Age, categorized into 1 to 12 years ii*)* Gender, categorized as male and female iii*)* Socioeconomic status of child’s family was calculated using Oyedeji’s formula [12]. This was further re-categorized as high, middle and low and iv*)* Body Mass Index (BMI), was calculated using their actual weight in kilogram and height in meters. This was categorized as underweight (<5th percentile), normal (5-85th percentile), overweight (85th–95th percentile) and obese (>95th percentile) based on their BMI for age and sex.

### Sample size calculation

The number of respondents enrolled in this study was calculated using the Cochran formula, N = f(α,β).2s^2^/(δ)^2^ for calculation of sample size based on a confidence interval of 95% which is equivalent to a confidence coefficient of 1.96.; where; N = minimum sample size; α = level of significance was taken to be 0.05; 1-β = power of the test (β was taken to be 0.9, so 1-β was calculated to be 0.1); δ = the smallest difference in means was taken to be 10%; s = standard deviation was taken to be 20 from a previous study done on age-based weight estimation by Eke et al. Computing the above variables, *N* = 10.5.2(20)^2^/(10)^2^ = 10.5 × 800/100 = 10.5 × 8 = 84. So, the minimum sample size will be 84 in each age group, giving a minimum sample size of 1008 children.

### Data collection and statistical analysis

Data collection was done using questionnaires administered by self and/or trained research assistants. Information were inputted into the relevant sections of the questionnaire and subsequently transferred into a Microsoft Excel Sheet. Statistical analysis was performed using SPSS (version 21; SPSS Inc., Chicago, IL USA) software.

To compare the weights estimated using Broselow tape and actual weight, the mean percentage error [100 x (estimated weight minus measured weight)/measured weight] and the absolute error (estimated weight minus measured weight) were calculated. A Bland-Altman plot was displayed to graphically present the bias and 95% limits of agreement. The percentage differences (errors) between estimated and measured weights were plotted on the y-axis while the averages of the two were on the x-axis. The dotted lines represent the limits of agreement (confidence interval) showing the degree of reliability while the spread of the scattered points depict the extent of agreement.

## Result

### Characteristics of study participants

A total of 1965 children between the ages of 1 to 12 years participated in this study. Of this number, 509 were excluded from analysis because their lengths were greater than 142 cm which is the length limit the Breslow-tape (BT) can measure. Consequently, 1456 were successfully enrolled and included in the final analysis. Table 1 shows that majority of the children (42%) surveyed were from families in the low socio-economic class while children from middle and high socio-economic class each made up about a third of the study participants. Majority (84%) of the study participants had body mass index (BMI) within normal z-score for age while male to female ratio was 0.9:1. Table 2 shows the mean difference and confidence interval between the estimated weight and measured weights.

**Table 1:** Summary statistics of children enrolled in this study

Study parameter	Variables	Frequency (n)	Percentage (%)
Age of respondents^a^ (*N* = 1456)	1 year	84	5.8
	2 years	104	7.1
	3 years	87	6.0
	4 years	68	4.7
	5 years	44	3.0
	6 years	120	8.2
	7 years	239	16.4
	8 years	222	15.2
	9 years	239	15.9
	10 years	174	12.0
	11 years	57	3.9
	12 years	25	1.7
Gender (*N* = 1456)	Male	669	45.9
	Female	787	54.1
Socio-economic class (*N* = 1455)	High	397	27.3
	Middle	439	30.2
	Low	619	42.5
BMI^b^ Z-Score Category (*N* = 1452)	Severe thinness	18	1.2
	Thinness	49	3.4
	Normal	1226	84.2
	Overweight	97	6.7
	Obesity	66	4.5

^a^Age at last birthday

^b^Body Mass Index

**Table 2:** Difference in mean weight between the actual measurement and Broselow-Tape estimation in each age category

Variables	Broselow estimation		Actual weight		Mean difference	Confidence interval
	N	Mean ± SD^a^	N	Mean ± SD	± SD	
Age category						
1 year	84	10.81 ± 1.18	84	10.57 ± 1.75	0.25 ± 1.10	−0.01, 0.48
2 years	104	13.62 ± 1.55	104	13.54 ± 1.97	0.08 ± 1.54	− 0.22, 0.38
3 years	87	15.75 ± 1.96	87	15.55 ± 2.39	0.21 ± 1.49	− 0.11, 0.53
4 years	68	18.68 ± 2.20	68	18.52 ± 2.77	0.17 ± 2.01	− 0.33, 0.66
5 years	44	20.52 ± 2.53	44	20.71 ± 3.36	−0.19 ± 2.15	−0.85, 0.46
6 years	120	23.80 ± 3.25	120	23.02 ± 3.75	0.78 ± 2.49	0.33, 1.23
7 years	239	26.64 ± 3.86	239	25.10 ± 4.56	1.54 ± 3.34	1.11, 1.96
8 years	222	28.61 ± 4.12	222	26.97 ± 4.97	1.63 ± 3.38	1.19, 2.08
9 years	232	31.16 ± 3.33	232	28.87 ± 4.74	2.29 ± 3.88	1.79, 2.79
10 years	174	32.10 ± 3.18	174	29.76 ± 4.45	2.34 ± 3.91	1.84, 2.84
11 years	57	33.19 ± 2.97	57	30.17 ± 4.74	3.02 ± 3.91	1.98, 4.06
12 years	25	33.44 ± 3.24	25	31.76 ± 5.42	1.68 ± 4.16	0.04, 3.40
Overall	1456	25.40 ± 7.55	1456	24.01 ± 7.30	1.39 ± 3.19	1.23, 1.56

^a^Standard deviation

### Accuracy and level of agreement between measured and estimated weights

Table 3 shows the mean percentage error (MPE) which is a measure of deviation of the weight estimates of the BT from the actual weights. It showed that the BT tended to overestimate weights in 1 year old by 3.88%, 2 years 1.58%, 3 years by 2.13%, 4 years (1.94%), 5 year (0.07%) and 6 years by 4.25%. After 6 years, the degree of overestimation rises sharply to 9.25% in 7, 7.29% in 8 and 9.29%. 9.18%, 11.61% & 6.75% in 9, 10, 11 and 12 years old respectively. Table 4 shows the accuracy of BT weight estimation that falls within 10 and 20% of the actual weight of surveyed children. It was seen that the proportion of Broselow tape weight estimations within 10% and 20% of the actual weight was higher in children in the 1 to 6-year groups with range of 64.7- 73.8% within 10% and 89.7- 96.7% within 20% agreement. The degree of agreement dropped significantly in the older age categories i.e. 40.0-59.0% and 77.2-88.3% respectively.

**Table 3:** Mean percentage error (MPE) and accuracy for estimated weights

Age variable	Mean Percentage Error (%)^a^	Confidence interval of MPE (%)		
		Standard deviation	Lower	Upper
1 year	3.88	12.42	- 20.46	28.22
2 years	1.58	11.48	- 20.92	24.08
3 years	2.13	9.49	- 16.47	20.73
4 years	1.94	10.98	−19.58	23.46
5 years	0.07	9.79	−19.12	19.25
6 years	4.25	10.48	−16.29	24.79
7 years	9.25	40.39	- 69.91	88.41
8 years	7.29	11.71	- 15.66	30.24
9 years	9.29	12.15	−14.52	33.10
10 years	9.18	12.51	- 15.34	33.70
11 years	11.61	13.54	- 14.93	38.15
12 years	6.75	11.67	- 16.12	29.62
Overall	6.67	19.75	- 32.04	45.38

^a^Positive value of MEP indicates overestimation of weight

**Table 4:** Agreement within 10 and 20% measured weight of estimated weight using the Breslow-Tape method

Age (years)	N	Estimated weight AGREEMENT with measured weight	
		Within ±10%	Within ±20%
1	84	62 (73.8)	77 (96.7)
2	104	70 (67.3)	96 (92.3)
3	87	61 (70.1)	82 (94.3)
4	68	44 (64.7)	61 (89.7)
5	44	31 (70)	41 (93.2)
6	120	80 (66.7)	113 (94.2)
7	239	141 (59.0)	211 (88.3)
8	222	119 (53.6)	185 (83.9)
9	232	100 (43.1)	191 (82.3)
10	174	89 (51.1)	196 (83.9)
11	57	24 (42.1)	44 (77.2)
12	25	10 (40.0)	22 (88.0)
Overall	1456	831 (57.1)	1269 (87.2)

### The Bland-Altman plot of Breslow-tape weight estimation

**Fig. 1:**
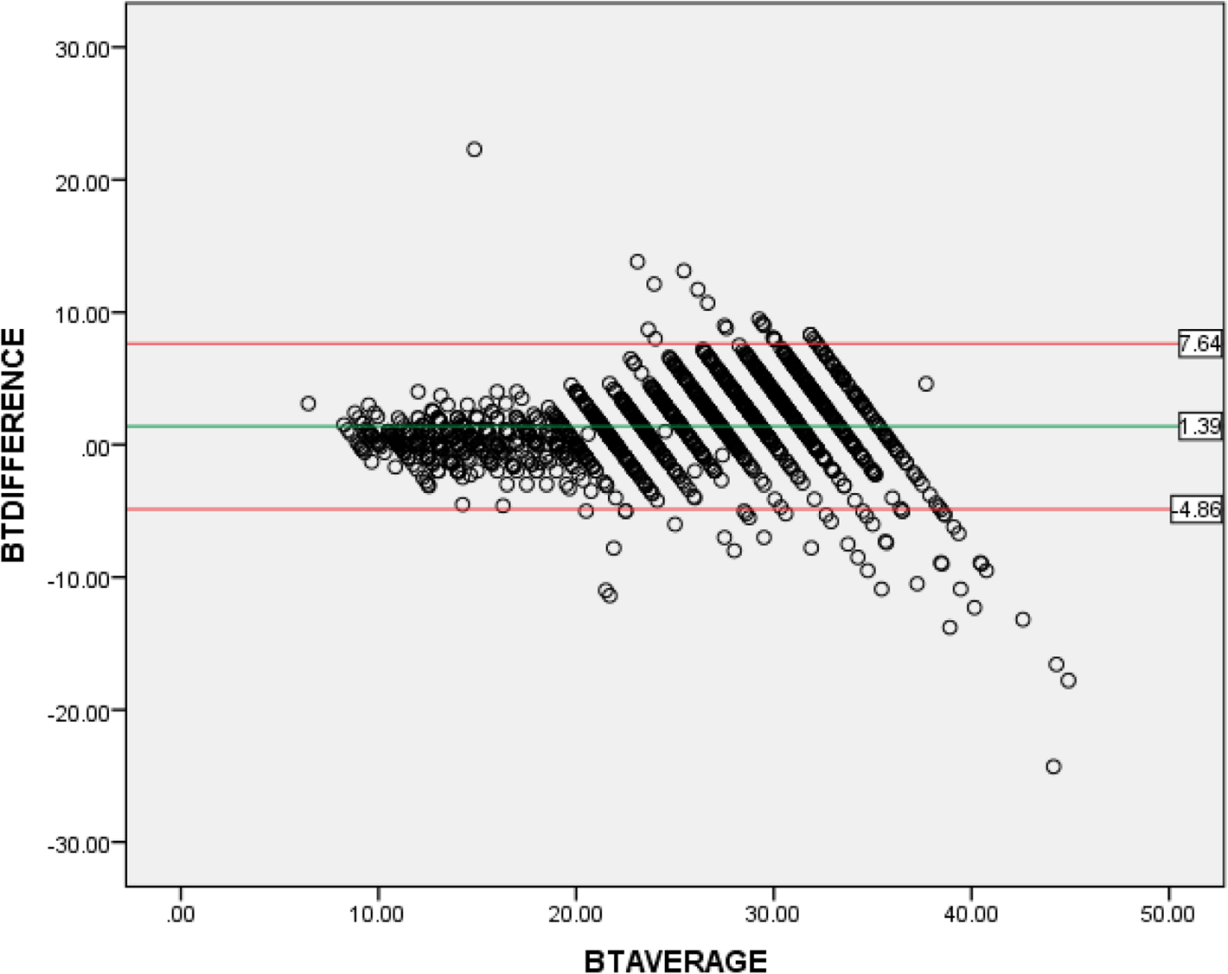
Bland-Altman plot of difference and average of measured and estimated weight using Breslow Tape for children aged 1–12 years

The Bland-Altman plot of the BT-weight estimation is shown in Fig. 1. It was noted that in the younger age categories, the estimated weights and actual weights were well clustered around the line of agreement. However, in the older age categories, the estimated weight was both above and below the line of agreement but well within the limits of agreement.

## Discussion

This study demonstrated a good correlation between the weight estimated by the BT and the actual weight of the children up to 6 years. Varghese et al. [13] in India and Gultekingil-keser et al. [14] in Turkey equally documented a good correlation of the Broselow tape with actual weight measurements in children. This however was not the case in the index study for children who were more than 6 years (or weighed >25kg) as their weights were overestimated with a mean percentage error of greater than 5% compared to less than 5% in younger ones.. Al Sulaibkh et al. [15] and Alharbi [16] and colleagues, both in Saudi Arabia observed similar occurrences that at weights of study participants of 26 kg and above, the Broselow tape overestimated the weights of the children. This suggests that both in low- and high-income countries, the Broselow tape does not correlate well with actual weights in children above 6 years of age.

A study done by Clark et al. [17] in South Sudan demonstrated inaccuracy of the Broselow tape measurements among their study participants irrespective of their nutritional status. South Sudan being a low-income country like Nigeria and in the same continent, did not show any correlation between the Broselow tape and the actual weight unlike in our own study where the tape accurately predicted the weight of children up to the age of 6 years. This disparity probably occurred because being a retrospective study, the researchers converted color measurements into zone for weight estimation rather than using the actual Broselow tape as done in the current study.

A systematic review and meta-analysis of the accuracy of the Broselow tape in estimating pediatric weight as well as its impact on drug calculation by Wells et al. [18] which reviewed 58 articles spanning from 1988 to 2017 showed it was most accurate for children between 10 and 25 kg and resulted in over 50% of children having a weight estimation within 10% of their actual body weight. The Broselow tape also consistently overestimates weight of children in low income countries while underestimating the weight of children in middle to high income countries. The findings documented by Wells et al. are comparable to the findings in the current study.

### Limitation

One major limitation of this study is in the categorization of children surveyed into age groups. The ages of these children were estimated based on the best recollection of their parents and/or caregivers. Due to logistic issues, we could not confirm the volunteered ages with their birth certificates. It is therefore possible that some undeterminable level of error in age estimation may have been introduced leading to misclassification into age groups and weights.

## Conclusion

Despite this limitation, our data which involved children from developing community showed that the Broselow tape measurements did not provide satisfactory results for all surveyed children. It was more reliable in children aged 1–6 years compared to those between 7–12 years. It is therefore recommended that childcare specialists be aware of these inaccuracies. Further research is required in other low and middle income countries to establish the accuracy of the Broselow tape before its application in the clinical setting of these countries.

## Data Availability

The datasets used and/or analyzed during the current study are available from the corresponding author on reasonable request.

## Abbreviations

BMI: Basal Metabolic Index
BT: Broselow tape
CI: Confidence interval
NHANES: National Health and Nutrition Examination Survey
OR: Odd ratio
